# Supplementary material for: Upper Limb Changes in DMD Patients Amenable to Skipping Exons 44, 45, 51 and 53: A 24-Month Study
Source: Children (Basel). 2023 Apr 19;10(4):746. doi: 10.3390/children10040746 (PMC10136754; doi:10.3390/children10040746)
Supplement: Supplementary file 1 [file children-10-00746-s001.zip › children-2265218-supplementary.pdf]

Supplementary Table S1. Details of number of assessments/patients included in the *Exon skipping class* analysis. *Key to table: Only completed data were used for the analysis. Patients with missing data at baseline or 24 months were excluded from the longitudinal analysis.*

	Number of patients	Number of paired assessments (baseline-24 months)
**Deletions**	**215**	**553**
**Duplications**	**26**	**60**
**Small mutations**	**70**	**195**
**Amenable to skip exon 44**	**27**	**66**
**Amenable to skip exon 45**	**25**	**59**
**Amenable to skip exon 51**	**34**	**103**
**Amenable to skip exon 43**	**24**	**76**

Supplementary Table S2. 12 and 24 months PUL changes subdivided in exon skip classes and ambulatory status. *= N represent the number of 24-months paired visits.

		AMBULANT				NON AMBULANT			
		**44 (N=48*)**	**45 (N=42*)**	**51 (N=48*)**	**53 (N=30*)**	**44 (N=18*)**	**45 (N=17*)**	**51 (N=55*)**	**53 (N=46*)**
**BASELINE**	**Age**								
	Mean (SD)	9.60 (4.30)	8.22 (2.18)	8.58 (2.29)	8.64 (3.72)	19.1 (5.19)	15.4 (4.14)	15.9 (4.06)	17.0 (4.64)
	Median [Min, Max]	8.53 [3.81, 22.1]	8.35 [3.80, 13.2]	8.36 [4.59, 13.6]	8.19 [4.12, 21.7]	20.3 [9.99, 26.5]	14.2 [10.0, 25.3]	15.7 [7.87, 25.7]	17.1 [8.83, 24.8]
	**TRANSITIONING**								
	NO	44 (91.7%)	37 (88.1%)	31 (64.6%)	18 (60.0%)	N/A	N/A	N/A	N/A
	YES	4 (8.3%)	5 (11.9%)	17 (35.4%)	12 (40.0%)	N/A	N/A	N/A	N/A
	**TOTAL PUL 2.0**								
	Mean (SD)	40.1 (1.51)	38.5 (3.17)	36.7 (4.51)	36.8 (5.01)	23.6 (8.43)	19.2 (10.3)	18.3 (9.25)	24.6 (9.83)
	Median [Min, Max]	40.0 [37.0, 42.0]	39.5 [29.0, 42.0]	37.5 [23.0, 42.0]	37.5 [24.0, 42.0]	22.5 [8.00, 38.0]	17.0 [3.00, 34.0]	16.0 [3.00, 39.0]	22.0 [10.0, 40.0]
**12 MONTH changes**	**TOTAL PUL 2.0**								
	Mean (SD)	0.104 (1.97)	-0.381 (4.06)	-0.458 (3.32)	0.0167 (3.18)	-2.75 (3.61)	-3.06 (3.12)	-2.33 (3.20)	-3.23 (4.06)
	Median [Min, Max]	0 [-7.00, 5.00]	0 [-17.0, 11.0]	0 [-8.00, 8.00]	-0.250 [-10.0, 8.00]	-1.50 [-12.0, 3.00]	-2.00 [-10.0, 2.00]	-2.00 [-13.0, 3.00]	-3.00 [-19.0, 3.00]
	**SHOULDER PUL 2.0**								
	Mean (SD)	0.0208 (1.35)	-0.214 (2.09)	-0.521 (2.02)	-0.200 (2.14)	-1.11 (1.74)	-1.29 (2.54)	-0.718 (1.67)	-1.39 (2.07)
	Median [Min, Max]	0 [-4.00, 3.00]	0 [-7.00, 6.00]	0 [-5.00, 4.00]	0 [-4.00, 6.00]	0 [-5.00, 0]	0 [-8.00, 0]	0 [-6.00, 3.00]	0 [-9.00, 0]
	**ELBOW PUL 2.0**								
	Mean (SD)	-0.0104 (0.789)	-0.274 (1.68)	-0.125 (1.67)	0.0667 (0.971)	-1.42 (1.70)	-1.03 (1.89)	-1.24 (1.84)	-1.63 (2.37)

	Median [Min, Max]	0 [-4.00, 1.00]	0 [-8.00, 4.00]	0 [-5.00, 5.00]	0 [-2.00, 3.00]	-1.00 [-6.00, 1.00]	-0.500 [-5.00, 2.00]	-1.00 [-7.00, 2.00]	-1.00 [-10.0, 2.00]
	**DISTAL PUL 2.0**								
	Mean (SD)	0.0938 (0.607)	0.107 (1.15)	0.188 (1.01)	0.150 (1.29)	-0.222 (1.23)	-0.735 (1.09)	-0.373 (1.07)	-0.207 (0.873)
	Median [Min, Max]	0 [-1.00, 1.00]	0 [-2.00, 5.00]	0 [-2.00, 3.00]	0 [-4.00, 3.00]	0 [-4.00, 2.00]	-0.500 [-3.00, 1.00]	0 [-3.00, 2.00]	0 [-3.00, 2.00]
**24 MONTH changes**	**TOTAL PUL 2.0**								
	Mean (SD)	-0.229 (2.58)	-1.71 (5.93)	-1.31 (4.82)	-1.67 (5.89)	-4.78 (4.35)	-5.41 (4.91)	-4.38 (3.81)	-6.39 (5.59)
	Median [Min, Max]	0 [-8.00, 5.00]	0 [-18.0, 11.0]	-1.00 [-12.0, 8.00]	-1.00 [-25.0, 11.0]	-3.50 [-13.0, 2.00]	-4.00 [-15.0, 1.00]	-4.00 [-16.0, 1.00]	-6.00 [-21.0, 1.00]
	**SHOULDER PUL 2.0**								
	Mean (SD)	-0.229 (1.49)	-1.26 (3.25)	-1.13 (2.83)	-1.27 (2.68)	-1.78 (2.65)	-2.35 (3.77)	-1.29 (2.39)	-2.57 (3.29)
	Median [Min, Max]	0 [-4.00, 3.00]	-0.500 [-9.00, 6.00]	0 [-7.00, 5.00]	-0.500 [-9.00, 4.00]	0 [-8.00, 0]	0 [-9.00, 0]	0 [-9.00, 0]	0 [-11.0, 0]
	**ELBOW PUL 2.0**								
	Mean (SD)	-0.146 (1.25)	-0.571 (2.37)	-0.417 (2.10)	-0.333 (2.66)	-2.44 (1.79)	-1.82 (2.21)	-2.33 (2.26)	-3.33 (3.00)
	Median [Min, Max]	0 [-5.00, 1.00]	0 [-8.00, 4.00]	0 [-5.00, 4.00]	0 [-10.0, 6.00]	-2.50 [-6.00, 0]	-2.00 [-6.00, 2.00]	-2.00 [-8.00, 1.00]	-3.00 [-12.0, 1.00]
	**DISTAL PUL 2.0**								
	Mean (SD)	0.146 (0.743)	0.119 (1.25)	0.229 (1.31)	-0.0667 (1.91)	-0.556 (1.69)	-1.24 (1.48)	-0.764 (1.35)	-0.500 (1.19)
	Median [Min, Max]	0 [-1.00, 2.00]	0 [-3.00, 5.00]	0 [-2.00, 4.00]	0 [-6.00, 6.00]	0 [-6.00, 2.00]	-1.00 [-4.00, 1.00]	-1.00 [-4.00, 3.00]	-1.00 [-4.00, 3.00]
